# Early Detection of Lower Adherence to Long‐Term e‐Diary Recording: A Checkpoint to Target Early Educational Intervention in Seasonal Allergic Rhinitis?

**DOI:** 10.1111/cea.70203

**Published:** 2026-01-23

**Authors:** S. Dramburg, C. J. Hernandez Toro, U. Grittner, S. Tripodi, S. Arasi, A. Acar Şahin, X. Aggelidis, A. Barbalace, A. Bourgoin, B. Bregu, M. A. Brighetti, E. Caeiro, S. Caglayan Sozmen, L. Caminiti, D. Charpin, M. Couto, L. Delgado, A. Di Rienzo Businco, C. Dimier, M. V. Dimou, J. A. Fonseca, O. Goksel, D. Hernandez, T. M. Hoffmann, D. T. Jang, F. Kalpaklioglu, B. Lame, R. Llusar, M. Makris, A. Mazon, E. Mesonjesi, A. Nieto, A. Öztürk, L. Pahus, G. Pajno, I. Panasiti, N. G. Papadopoulos, E. Pellegrini, S. Pelosi, A. M. Pereira, M. Pereira, N. M. Pinar, E. Potapova, A. Priftanji, F. Psarros, C. Sackesen, I. Sfika, J. Suarez, M. Thibaudon, A. Travaglini, U. Uguz, V. Verdier, V. Villella, P. Xepapadaki, D. Yazici, P. M. Matricardi

**Affiliations:** ^1^ Department of Pediatric Respiratory Care, Immunology and Intensive Care Medicine Charit—Universitätsmedizin Berlin Berlin Germany; ^2^ Institute of Biometry and Clinical Epidemiology Charité—Universitätsmedizin Berlin Berlin Germany; ^3^ Pediatric Allergy Unit Sandro Pertini Hospital Rome Italy; ^4^ Allergolology Service, Policlinico Casilino Rome Italy; ^5^ Pediatric Allergology Unit, Department of Pediatric Medicine Bambino Gesù Children´s Research Hospital (IRCCS) Rome Italy; ^6^ Department of Biology, Faculty of Science Ankara University Ankara Turkey; ^7^ Allergy Unit, 2nd Department of Dermatology and Venereology National and Kapodistrian University of Athens, University Hospital ‘Attikon’ Athens Greece; ^8^ Department of Pediatrics—Allergy Unit University of Messina Messina Italy; ^9^ Department of Pneumonology and Allergy La Timone Hospital, APHM, Aix‐Marseille University Marseille France; ^10^ Department of Allergology and Clinical Immunology UHC Mother Teresa, Medical University Tirana Tirana Albania; ^11^ Department of Biology Tor Vergata University Rome Italy; ^12^ MED‐ Mediterranean Institute for Agriculture, Environment and Development & CHANGE—Global Change and Sustainability Institute, Institute for Advanced Studies and Research University of Évora Évora Portugal; ^13^ Portuguese Society of Allergology and Clinical Immunology Lisbon Portugal; ^14^ Fenerbahce University Istanbul Turkey; ^15^ Immunoallergology, Hospital CUF Trindade Porto Portugal; ^16^ Basic and Clinical Immunology Unit, Department of Pathology, Faculty of Medicine University of Porto Porto Portugal; ^17^ RISE‐Health Faculty of Medicine of the University of Porto Porto Portugal; ^18^ Allergy Unit Instituto and Hospital CUF Porto Porto Portugal; ^19^ Allergy Department, 2nd Pediatric Clinic, Athens General Children's Hospital ‘P&A Kyriakou’ University of Athens Athens Greece; ^20^ Department of Pulmonary Medicine, Division of Immunology, Allergy and Asthma, Faculty of Medicine, EgeSAM‐Ege University Translational Pulmonary Research Center Ege University Izmir Turkey; ^21^ Department of Allergy Health Research Institute Hospital La Fe Valencia Spain; ^22^ Pediatric Allergy and Pneumology Unit Children's Hospital La Fe, Health Research Institute La Fe Valencia Spain; ^23^ Department of Immunology and Allergic Diseases Kırıkkale University School of Medicine Kırıkkale Turkey; ^24^ Department of Pulmonary Medicine, Division of Allergy and Immunology, School of Medicine Koç University Istanbul Turkey; ^25^ Aix Marseille Univ, APHM, INSERM CIC 1409, INSERM U1263, INRA 1260 (C2VN), Marseille France; ^26^ Division of Infection, Immunity & Respiratory Medicine, Royal Manchester Children's Hospital University of Manchester Manchester UK; ^27^ Department of Reggio Calabria ARPA‐ Regional Agency for Environmental Protection Calabria Italy; ^28^ TPS Production Srl Rome Italy; ^29^ Allergy Department Athens Naval Hospital Athens Greece; ^30^ Division of Pediatric Allergy Koç University School of Medicine Istanbul Turkey; ^31^ Department of Biology of Organisms and Systems, Area of Botany University of Oviedo Oviedo Spain; ^32^ Réseau National de Surveillance Aérobiologique Brussieu France; ^33^ Italian Aerobiology Monitoring Network—Italian Aerobiology Association Rome Italy; ^34^ Department of Biology, Faculty of Science Ege University Izmir Turkey; ^35^ Allergy Department, 2nd Pediatric Clinic National and Kapodistrian University of Athens Athens Greece; ^36^ Cellular and Molecular Medicine, KUTTAM, Graduate School of Health Sciences Koç University Istanbul Turkey; ^37^ Swiss Institute of Allergy and Asthma Research (SIAF) University of Zurich Davos Switzerland; ^38^ Institute of Allergology Charité—Universitätsmedizin Berlin, Corporate Member of Freie Universität Berlin and Humboldt‐Universität Zu Berlin Berlin Germany; ^39^ Fraunhofer Institute for Translational Medicine and Pharmacology ITMP, Allergology and Immunology Berlin Germany

**Keywords:** adherence, allergic rhinitis, allergy symptoms, chronic diseases, e‐diary, patient education, patient‐reported outcomes, remote monitoring, reporting behaviour

## Abstract

**Background:**

Digital symptom monitoring via e‐Diary apps can support the diagnosis and management of chronic diseases with trigger‐induced exacerbations such as pollen allergies. Attrition is a major challenge for continuous e‐Diary usage with an unsupervised approach.

**Objective:**

To investigate adherence to e‐Diary reporting, its early determinants and predictors in a blended care setting among pollen allergic patients with heterogeneous cultural backgrounds.

**Methods:**

The @IT.2020 observational multicenter study recruited patients with diagnosed seasonal allergic rhinitis from seven Southern European/Mediterranean countries. Baseline characteristics were investigated through questionnaires, skin prick tests and serum specific IgE measurements. The study doctors asked patients to record their allergy symptoms via e‐Diary (AllergyMonitor, TPS) daily during the clinically relevant season of pollination and increased mould concentrations.

**Results:**

Among 815 patients (467 adults, 348 children), the average prescribed e‐Diary recording period was 106 (SD 47.1) days, with an average completion rate of 75.2% (SD 21.2%). Children (≥ 10 years) filled 73.8% (95% CI 68.1–79.4) of prescribed days without parental support. We identified a stable ‘higher’ and a more variable ‘lower’ adherence cluster. Adherence was weakly associated with disease severity, but not with age, gender, country, education or digital literacy. Short‐term (first 3 weeks) adherence was strongly associated with long‐term adherence (partial *R* ^2^ = 0.387, *p* < 0.001), with 87.6% of lower adherent patients remaining poorly adherent beyond 3 weeks.

**Conclusion:**

In a blended care setting, adherence to e‐Diary compilation among pollen allergic patients is high, irrespective of age and cultural background. Early identification of lower adherence is possible and might inform early interventions to improve patient adherence.

## Key Messages

- Adherence to e‐Diary is high (75.2% average) among pollen allergic patients in a blended care setting.
- Two clusters of patients identified: one with stable ‘higher’ and one with variable ‘lower’ adherence.
- Low adherence can be identified early (after 3 weeks only), opening chances for early intervention.

## Graphical abstract

Adherence to e‐Diary reporting was investigated in a blended care setting among pollen allergic patients with heterogeneous cultural backgrounds. A stable ‘higher’ and a more variable ‘lower’ adherence cluster were identified. Early identification of lower adherence is possible and might inform early interventions to improve patient adherence.

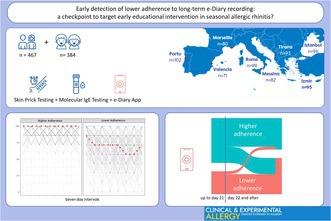

**Abbreviations**
AR: allergic rhinitis
ARIA: Allergic rhinitis and its impact on asthma
ATH: Study centre in Athens, Greece
DHI: Digital Health Intervention
DHLI: Digital Health Literacy Instrument
e‐CRF: electronic clinical record form
e‐Diary: Electronic diary
IST: Study centre in Istanbul, Turkey
IQR: Interquartile range
IZM: Study centre in Izmir, Turkey
LMM: Linear Mixed Model
MAR: Study centre in Marseille, France
MES: Study centre in Messina, Italy
mHealth: mobile Health
NCD: Non‐communicable disease
POR: Study centre in Porto, Portugal
RCT: Randomised controlled trial
ROM: Study centre in Rome, Italy
SAR: Seasonal allergic rhinitis
SD: Standard deviation
SMD: Standardised mean difference
SPT: Skin prick test
SUS: System Usability Scale
TIR: Study centre in Tirana, Albania
VAL: Study centre in Valencia, Spain
VAS: Visual analogue scale

## 1 Introduction

Mobile health (mHealth) interventions have become a popular tool in the self‐management of non‐communicable diseases (NCD) [1, 2, 3]. In particular, the usage of electronic diaries (e‐Diary) to monitor chronic diseases with trigger‐induced exacerbations, such as migraines [4, 5], asthma [6, 7] or allergic rhinitis (AR) [8, 9, 10] has been highly investigated. Thanks to the broad access to smartphone technology and increasing internet coverage, e‐Diaries are easily accessible and scalable tools for different age groups and diseases, both relevant aspects for the implementation of digital health interventions (DHI) in large patient populations.

Unfortunately, non‐adherence to DHI can be an important limitation for their implementation across several health domains [11, 12]. In clinical studies with short (< 1 month) to medium (3 months) duration with additional intervention and regular contact with the study staff, e‐Diaries have shown to be reliable and patients are highly adherent to filling the asked dataset [13, 14]. However, a recent systematic review, summarising different studies concerning a range of NCD, showed that users of mHealth apps covered on average only 53.4% (SD 24.7%) of the intended app interactions [15]. In the area of respiratory diseases, app characteristics such as design and usability, and personal contact with a healthcare professional were identified as factors positively associated with mHealth adherence [15].

The relevance of a close patient‐doctor partnership in e‐Diary compilation (‘blended care concept’) also emerges from studies on remote monitoring of seasonal allergic rhinitis (SAR) and asthma [16, 17, 18]. Spontaneous downloads and uses of the validated e‐Diary app MASK‐Air for AR and asthma were associated with a decrease in adherence down to 7.9% after only 1 week of recording despite high numbers of users (> 58.000) [16]. By contrast, we observed in a previous study that the doctor‐prescribed download and use of a similar, validated e‐Diary app AllergyMonitor resulted in an adherence of around 80% over three months [17]. During that study, 101 Italian children and 99 adults with seasonal allergic rhinitis (SAR) had at least two personal contacts with a study physician. In addition to automated reminders, patients received a personalised E‐Mail and/or phone call from the study nurse whenever more than 3 consecutive days of reporting were missed [17]. Therefore, we assumed that doctor's prescription and personal contact are the best strategy for a successful implementation of mobile monitoring tools, such as e‐Diaries [17].

To test the generalizability of our assumption, we repeated the investigation within the multicenter @IT.2020 observational study, implemented in nine different geographical and cultural settings across the Mediterranean region. While the overall aim of the project is to assess the impact of mHealth technologies and molecular specific IgE diagnostics on the diagnosis of pollen allergies [19, 20], the present analysis is focused on the e‐Diary reporting behaviour of pollen‐allergic patients, specifically the adherence to e‐Diary reporting and its early determinants and predictors.

## 2 Material and Methods

### 2.1 Study Population, Design and Setting

The fieldwork of the multicentre phase of the @IT.2020 observational study was performed between December 2017 and October 2018 in 9 Southern European allergy clinics: Porto (Portugal), Valencia (Spain), Marseille (France), Rome, Messina (both Italy), Tirana (Albania), Athens (Greece), Istanbul and Izmir (both in Turkey). Patients, aged 10–65 years with a confirmed diagnosis of SAR, were recruited to attend two study visits (T0 inclusion visit and T1 after the end of the pollen season 2018) and complete a monitoring period in between the two visits. During the individually prescribed monitoring period, participants were asked to fill a daily electronic symptoms and medication diary via the app AllergyMonitor (TPS Production Srl, Rome, Italy). The study was approved by the local ethics committees: NNA Scientific Council (13,631/09.10.2017), EGE University Rectorate Clinical Research Ethics Committee (17–7.1/21), CPP NORD OUEST III (2017‐A03364‐49), Comitato Etico Messina (17577), Republic of Albania Ethics Commission (405/5), Committee on Human Research (568–4), Comissão de Ética (389/2017), Comitato Etico Lazio 2 (2673), CEBCI (39,524/2017). All participants, or their legal guardians, provided written informed consent.

Details on the study protocol of the @IT.2020 pilot and multicenter study can be found in the online repository and have been previously published [17, 19, 20].

### 2.2 AllergyMonitor E‐Diary App

The e‐Diary app AllergyMonitor is a digital platform combining a mobile patient app with a browser‐based back‐office for healthcare professionals. The e‐Diary has been used and validated in several scientific studies [10] focused on data completeness [17] and validity [21, 22], drug self‐administration [23], and reliability of different symptom (medication) scores to assess AR severity [24, 25]. Briefly, the patient app allows daily recording of ocular, nasal and pulmonary symptoms responding to standardised questions. More detailed information on the daily questionnaires can be found in the online repository.

### 2.3 Definitions for Adherence to Symptom Reporting

To describe the reporting behaviour of our cohort, we propose a uniform terminology [17]. Briefly, adherence in the prescribed period refers to the days with a complete record (%) during the prescribed timeframe (all days fixed by the attending physician), while adherence in the reporting period was calculated as the days with a complete record (%) during the reporting period (which excludes the delayed reporting start and anticipated reporting end) (Figure e1). If not stated otherwise, the term ‘adherence’ refers to adherence in the prescribed period. More detailed information on the daily questionnaires can be found in the online repository.

### 2.4 Statistics

Categorical data was summarised as counts and frequencies and continuous data was summarised as mean and standard deviation or as median, first, and third quartiles. To provide a standardised effect size of centre differences for baseline and app records descriptors, the mean Standardised Mean Difference (SMD) of pairwise comparisons between study centres was estimated. The SMD is Cohen's d for continuous normally distributed data and was calculated for binary and categorical data as proposed by Austin 2009 [26] and Yang and Dalton 2012 [27] respectively.

The percentage of postponed reporting was calculated as the percentage of all reported days by a patient for which the input date came after the date associated with the reported symptoms. An additional analysis comparing the adherence between groups of paediatric patients based on who filled most e‐Diary reports is described in the Supporting Information S1.

As adherence was not constant over time, unsupervised clustering analyses around medoids (a more robust version of k‐means) [28] were performed to find temporal patterns (per‐7‐day‐segment adherence) in the first 21 days of the prescribed period (‘early period’) and in the interval between Day 22 and Day 56 (‘later period’). For more details, see Supporting Information S1.

The association between short‐term (between 1st and 21st day of the prescribed period) and long‐term (after 21st day) adherence was evaluated with a linear mixed model (LMM) with random intercept by study centre. The dependent variable was individual long‐term adherence and independent variables were individual short‐term adherence and baseline characteristics (age, gender, AR ARIA classification at T0, length of prescribed period, the top 4 most frequent allergic comorbidities, years of schooling, education level, Digital Health Literacy Instrument (DHLI), and previous use of health apps). Spearman and Pearson correlations between short‐term and long‐term adherence, as well as partial *R* ^2^ for the covariates in the linear mixed model, were estimated.

All statistical analyses were conducted using R (version 4.2.2) (http://www.r‐project.org/). A detailed list of the R libraries is available in Supporting Information S1.

The manuscript adheres to the STROBE statement to provide clear reporting [29].

## 3 Results

### 3.1 Study Population

The study cohort comprised 815 patients (467 adults and 348 children and adolescents), of which 700 attended the final study visit and 760 reported symptoms via the study app. Allergic asthma (168/815, 20.6%), atopic dermatitis (179/815, 22%) and urticaria/angioedema (187/815, 22.9%) were the most common allergic comorbidities (Table e1). A great heterogeneity of IgE sensitization profiles was observed and described in detail elsewhere [22]. More information on baseline characteristics can be found in the online repository.

### 3.2 Digital Health Literacy and Previous Use of Health Apps

The ability to retrieve health‐related information online and estimate its scientific quality was high across the adult study population with a mean score of 3.3 (SD: 0.4) in the DHLI (maximum score = 4points). Although a similarly high score was reached among children and adolescents (3.2; SD: 0.5), there was more inter‐center variation for this age group (Table e1). Three‐quarters of both the paediatric and the adult study population subsets reported having previously used a smartphone application; the use of health apps being slightly more frequent among adults than children (27.4% [88/321, 95% CI = 22.6% to 32.6%] vs. 24.1% [71/294, 95% CI = 19.4% to 29.4%]) (Table e1). Remarkably, 243/321 (75.7%) of the adults and 227/294 (77.2%) of the children/adolescents denied the previous use of health‐related mobile applications (Table e1).

### 3.3 Adherence to e‐Diary Symptom Reporting

The shortest prescribed period of symptom reporting was 58, the longest 212 days. Accordingly, the mean length of prescribed reporting per patient was heterogeneous between study centres (SMD = 0.77), ranging between 69.8 (SD: 22.9) days in Messina and 142.2 (SD: 39.0) days in Rome (Table 1). The average adherence during the prescribed monitoring period was 75.2% (SD: 21.2%) with low variations between centres (SMD 0.28). The percentage of postponed reporting (retrospective recording for the previous day) by patient ranged between 37.7% (SD: 18.2%) in Marseille and 46.3% (SD: 20.4%) in Messina (Table 1). Missing values were most frequently observed as gaps of 1 day (on average 6.2% of the prescribed days), followed by 5 days or more of non‐recording in 4.8% (SD: 9.5%). One‐day gaps were most prevalent in Istanbul, Marseille, Messina, Porto, Rome, and Tirana, while in Athens, Izmir, and Valencia most missing days were part of 5‐day intervals or longer (Table 1). The AllergyMonitor app was evaluated as easy and intuitive to use in all study centres. The average rating of the System Usability Scale (SUS—maximum score = 100 points) across all centres was 79.2 points (SD: 15.6), ranging from 70.7 (SD 17.4) in Athens to 85.9 (SD: 16.9) in Tirana (Table 1).

**TABLE 1:** Description of the e‐Diary reports across centres.

	ATH	IST	IZM	MAR	MES	POR	ROM	TIR	VAL	Overall	Mean SMD
	*n* = 90	*n* = 94	*n* = 92	*n* = 77	*n* = 81	*n* = 101	*n* = 99	*n* = 93	*n* = 33	*n* = 760	
Who filled the eDiary mostly? *n* (%)^a^											0.96
Adult patient	37 (47.4)	43 (65.2)	43 (47.8)	75 (100.0)	30 (46.2)	42 (50.0)	39 (48.8)	65 (72.2)	0 (0.0)	374 (56.7)	
Paediatric patient	26 (33.3)	21 (31.8)	24 (26.7)	0 (0.0)	33 (50.8)	36 (42.9)	36 (45.0)	17 (18.9)	24 (75.0)	217 (32.9)	
Parent or guardian	8 (10.3)	0 (0.0)	6 (6.7)	0 (0.0)	2 (3.1)	2 (2.4)	3 (3.8)	5 (5.6)	7 (21.9)	33 (5.0)	
Both Ped. Pat & Par/Guard	7 (9.0)	2 (3.0)	17 (18.9)	0 (0.0)	0 (0.0)	4 (4.8)	2 (2.5)	3 (3.3)	1 (3.1)	36 (5.5)	
System Usability Score; mean (SD)^b^	70.7 (17.4)	77.5 (12.1)	81.1 (16.3)	—	77.0 (16.7)	84.3 (11.2)	78.0 (11.3)	85.9 (16.0)	75.1 (18.0)	79.2 (15.6)	0.40
Prescribed reporting days per patient; mean (SD)	129.3 (44.6)	78.9 (29.0)	102.3 (45.7)	92.0 (33.3)	69.8 (22.9)	102.5 (52.3)	142.2 (39.0)	129.1 (48.3)	89.4 (36.6)	106.0 (47.1)	0.77
Recorded days per patient; mean (SD)	88.8 (42.8)	59.5 (26.2)	78.6 (42.8)	69.8 (31.5)	49.3 (22.9)	73.1 (45.2)	115.1 (41.2)	105.5 (45.1)	58.0 (24.6)	79.8 (43.3)	0.73
Percentage of records within the prescribed period per patient; mean (SD)	69.1 (23.1)	75.8 (19.5)	76.8 (20.7)	76.2 (19.9)	70.6 (22.4)	70.9 (22.9)	81.3 (19.2)	82.0 (16.1)	69.9 (24.4)	75.2 (21.2)	0.28
Percentage of records within the reporting period per patient; mean (SD)	75.2 (17.1)	78.8 (17.0)	78.8 (19.2)	80.6 (15.3)	74.6 (19.3)	74.6 (20.1)	85.6 (12.6)	85.3 (12.9)	76.4 (20.0)	79.1 (17.5)	0.30
Percentage of days from prescribed period covered by the reporting period; mean (SD)	90.0 (17.9)	95.4 (11.0)	96.5 (10.4)	93.2 (14.3)	93.2 (13.6)	94.3 (12.9)	94.7 (16.2)	95.7 (9.8)	89.5 (16.2)	94.0 (13.7)	0.20
Percentage of days missing from prescribed period due to late start; mean (SD)	0.8 (2.3)	1.0 (3.9)	1.2 (3.6)	1.8 (6.8)	1.7 (5.2)	1.4 (2.8)	0.5 (1.7)	1.0 (2.8)	0.5 (1.0)	1.1 (3.8)	0.17
Percentage of days missing from prescribed period due to early stop; mean (SD)	9.2 (17.7)	3.6 (9.8)	2.2 (9.5)	5.0 (11.8)	5.1 (11.8)	4.3 (11.6)	4.8 (15.5)	3.3 (9.6)	10.0 (16.2)	4.9 (12.8)	0.22
Percentage of recorded days with postponed recording per patient; mean (SD)	43.8 (15.8)	45.6 (19.3)	43.2 (19.8)	37.7 (18.2)	46.3 (20.4)	39.0 (25.4)	38.3 (20.2)	41.1 (18.0)	42.4 (17.0)	41.9 (19.9)	0.19
Percentage missing days due to: mean (SD)											
Gaps of 1 day	5.0 (3.2)	7.1 (4.9)	5.6 (4.0)	5.4 (3.9)	7.3 (5.2)	7.7 (5.3)	5.3 (3.9)	6.2 (4.2)	5.5 (3.8)	6.2 (4.5)	0.26
Gaps of 2 days	3.9 (3.9)	5.1 (5.3)	4.3 (4.2)	3.8 (4.4)	4.6 (5.4)	5.5 (5.8)	2.7 (3.0)	3.1 (3.5)	3.5 (4.4)	4.1 (4.6)	0.25
Gaps of 3 days	2.0 (2.7)	2.0 (3.3)	2.4 (4.3)	2.3 (3.4)	3.5 (4.4)	2.8 (4.8)	1.3 (2.0)	1.0 (1.9)	2.7 (3.4)	2.2 (3.6)	0.27
Gaps of 4 days	1.9 (2.9)	1.5 (3.6)	1.7 (3.3)	1.8 (3.6)	2.0 (4.1)	2.0 (3.7)	1.1 (2.2)	0.8 (1.9)	1.7 (3.8)	1.6 (3.2)	0.16
Gaps of 5 days or more	8.1 (9.8)	4.0 (8.8)	5.8 (12.5)	3.6 (7.3)	5.2 (10.8)	5.3 (10.1)	3.0 (5.9)	2.7 (7.3)	6.1 (11.0)	4.8 (9.5)	0.22

^a^ *n* = 660; the information on who recorded the eDiary was not available for all participants who completed the diary.

^b^ *n* = 576; Not all participants who filled the eDiary filled the SUS questionnaire.

**FIGURE 1:**
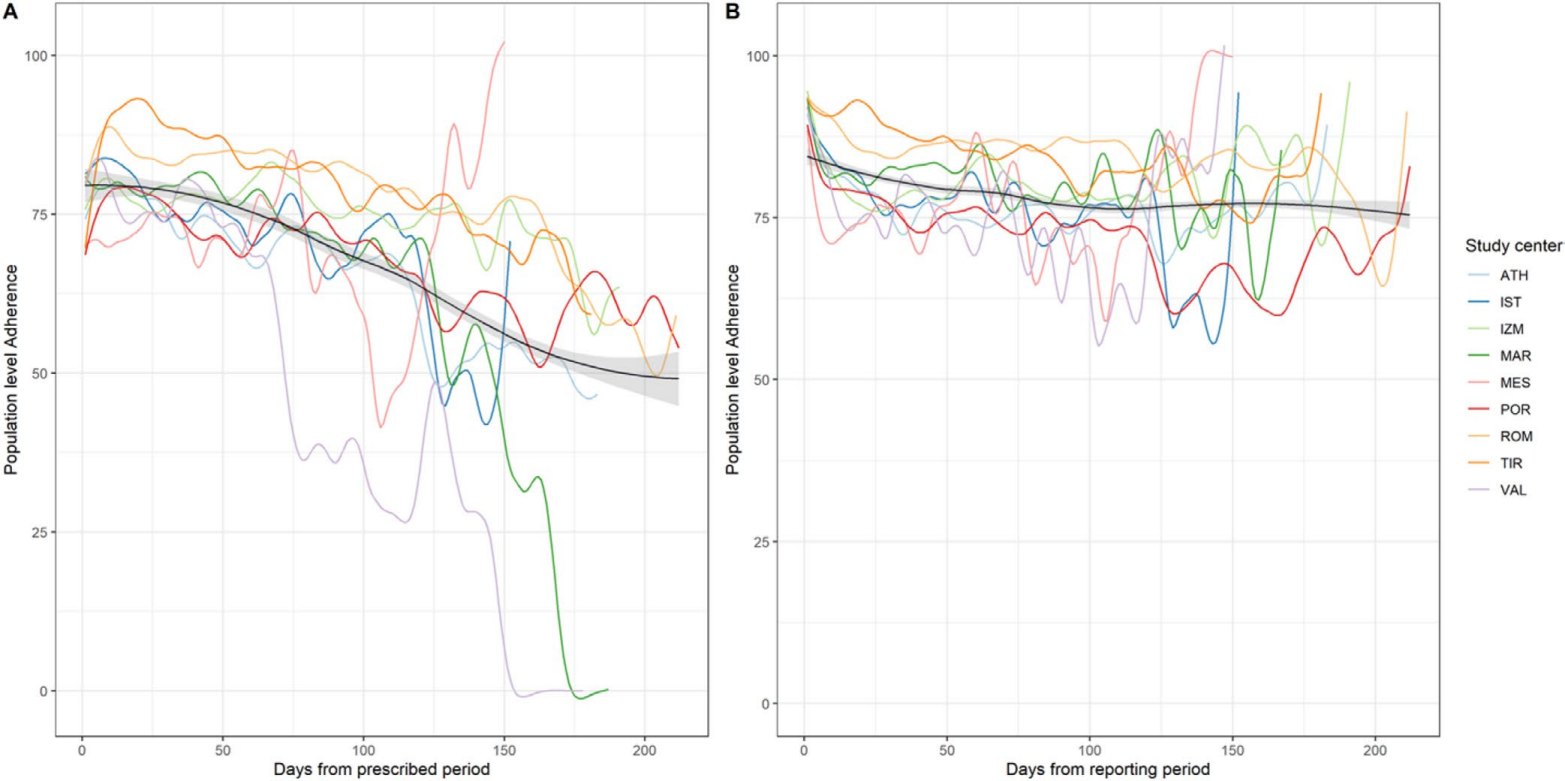
Population level adherence to symptom recording in study app (*N* = 760) per centre within (A) prescribed periods and (B) reporting periods. Adherence in the prescribed period is the adherence (%) of days during the prescribed timeframe for which patients reported their symptoms; adherence in the reporting period was calculated as the adherence (%) of days in the reporting period for which patients reported their symptoms. If not stated otherwise, the term ‘adherence’ refers to adherence in the prescribed period. Curves were smoothed with spline models with 25 degrees of freedom. Overall locally estimated scatterplot smoothing (LOESS) trend line (black) and its standard error are shown. ATH, Athens, IST, Istanbul, IZM, Izmir, MAR, Marseille, MES, Messina, POR: Porto, ROM, Rome, TIR, Tirana, VAL, Valencia.

Overall, the population level adherence remained high in most centres for both the prescribed period and the reporting period (Figure 1). In both cases, during the first 60 days of the reporting period the population level adherence was above 75% and for some centres around the 100th day dropped below ~60% (Figure 1B).

We observed large differences between study centres regarding the self‐reported information on who recorded the e‐Diary for most of the time, patients, parents/guardians, or both (mean SMD = 0.96) (Table 1). Noteworthy, parents or guardians recorded symptoms for the participating child/adolescent in only 33/286 cases (11.5%), while 217/286 (75.9%) of children did the recording independently from their parents/guardians. The estimated average adherence of paediatric patients alone was almost as high as when parents/guardians filled the e‐Diary, with an adherence of 73.8% (95% CI = 68.1% to 79.4%) and 81.4% (95% CI = 73.1% to 89.8%), respectively. When parents and children shared the recording, the estimated adherence was 71.0% (95% CI = 62.6% to 79.3%) (Table e2).

### 3.4 Association Between Short‐Term Reporting Behaviour and Long‐Term Adherence

**FIGURE 2:**
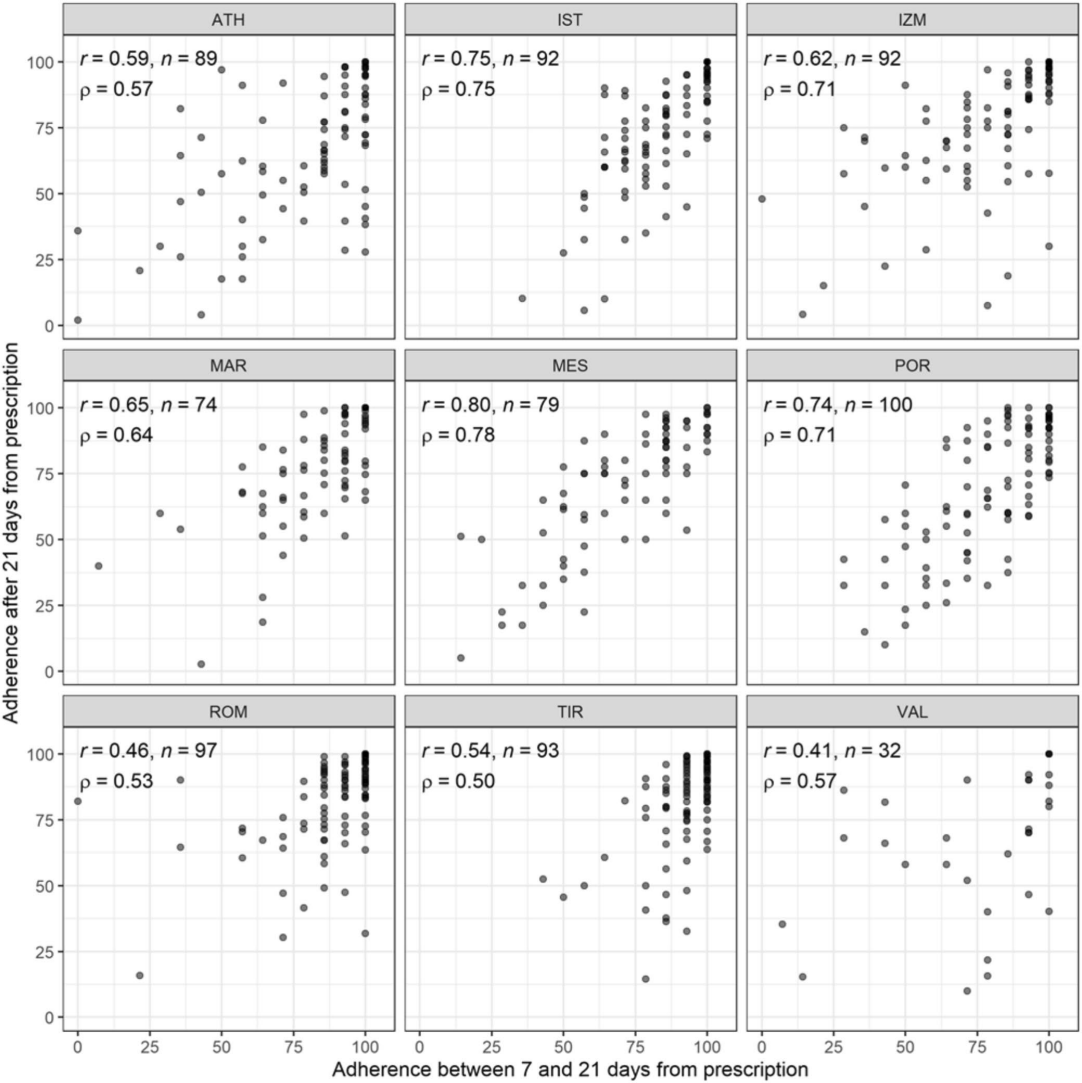
Association between short‐term (between Day 7 and Day 21) and long‐term adherence (after Day 21) per study centre. Pearson and Spearman's correlation and total number of patients included per centre are shown. The excluded patients stopped symptom recording before Day 21 of their prescribed period.

To evaluate the association between short‐ and long‐term adherence, we estimated Pearson (r) and Spearman (ρ) correlation between the two for each centre. In all centres, a positive correlation between both adherences was observed (Figure 2). To identify associations between the long‐term adherence, previous reporting behaviour (short‐term adherence), and a patient's baseline characteristics we fitted a linear mixed model with random intercept by study centre and the variables of interest as covariates. In this model, we found no existence of substantial associations between long‐term adherence and any of the baseline characteristics considered except for the age of the patient and the ARIA classification at T0, both of which had small effect sizes (Table 2). Additionally, short‐term adherence had a larger effect size than all the considered baseline characteristics, while centre had little impact on the long‐term reporting behaviour of patients (Table 2).

**TABLE 2:** Estimated contribution of baseline characteristics and short‐term reporting behaviour to long‐term reporting behaviour from linear mixed model: Long‐term adherence on short‐term adherence and baseline characteristics with random intercept by study centre.

	Estimates	CI	*p*	Effect size
*Fixed effects*				
Model intercept	17.07	2.22–31.91	**0.024**	—
Short term reporting adherence (per 1% increase)	0.68	0.61–0.75	**< 0.001**	0.382
Age (per 1‐year increase)	0.16	0.01–0.31	**0.042**	0.007
Gender				
Female	Reference			
Male	−0.49	−3.40–2.43	0.744	< 0.001
ARIA classification (T0)				
Mild‐intermittent	Reference			
Severe‐intermittent	5.31	−2.03–12.65	0.156	0.004
Mild‐persistent	10.41	2.29–18.52	**0.012**	0.011
Severe‐persistent	7.40	0.65–14.14	**0.032**	0.008
Comorbidities				
Allergic Asthma	3.45	−0.15–7.06	0.060	0.006
Atopic Dermatitis	0.14	−3.55–3.83	0.941	< 0.001
Urticaria	−1.99	−5.64–1.67	0.286	0.002
Oral Allergy Syndrome	0.19	−4.03–4.40	0.930	< 0.001
Years of schooling (per 1‐year increase)	−0.07	−0.86–0.73	0.872	< 0.001
Length of prescribed period (per 1‐day increase)	−0.01	−0.04–0.02	0.500	0.001
Education level				
Primary school	Reference			
Secondary school	2.78	−1.99–7.56	0.252	0.003
Professional formation	−2.54	−10.21–5.13	0.516	0.001
University degree	1.79	−6.54–10.12	0.674	< 0.001
Previous use of health apps (Yes)	0.03	−3.27–3.33	0.988	< 0.001
Digital Health Literacy Index (per 1‐point increase)	−2.44	−5.69–0.81	0.141	0.004
*Random effects*				
ICC	0.03			
*N* centre	8			
Observations	568			
Marginal *R* ^2^/Conditional *R* ^2^	0.413/0.428			

### 3.5 Adherence Clusters Over Time and Their Determinants

**FIGURE 3:**
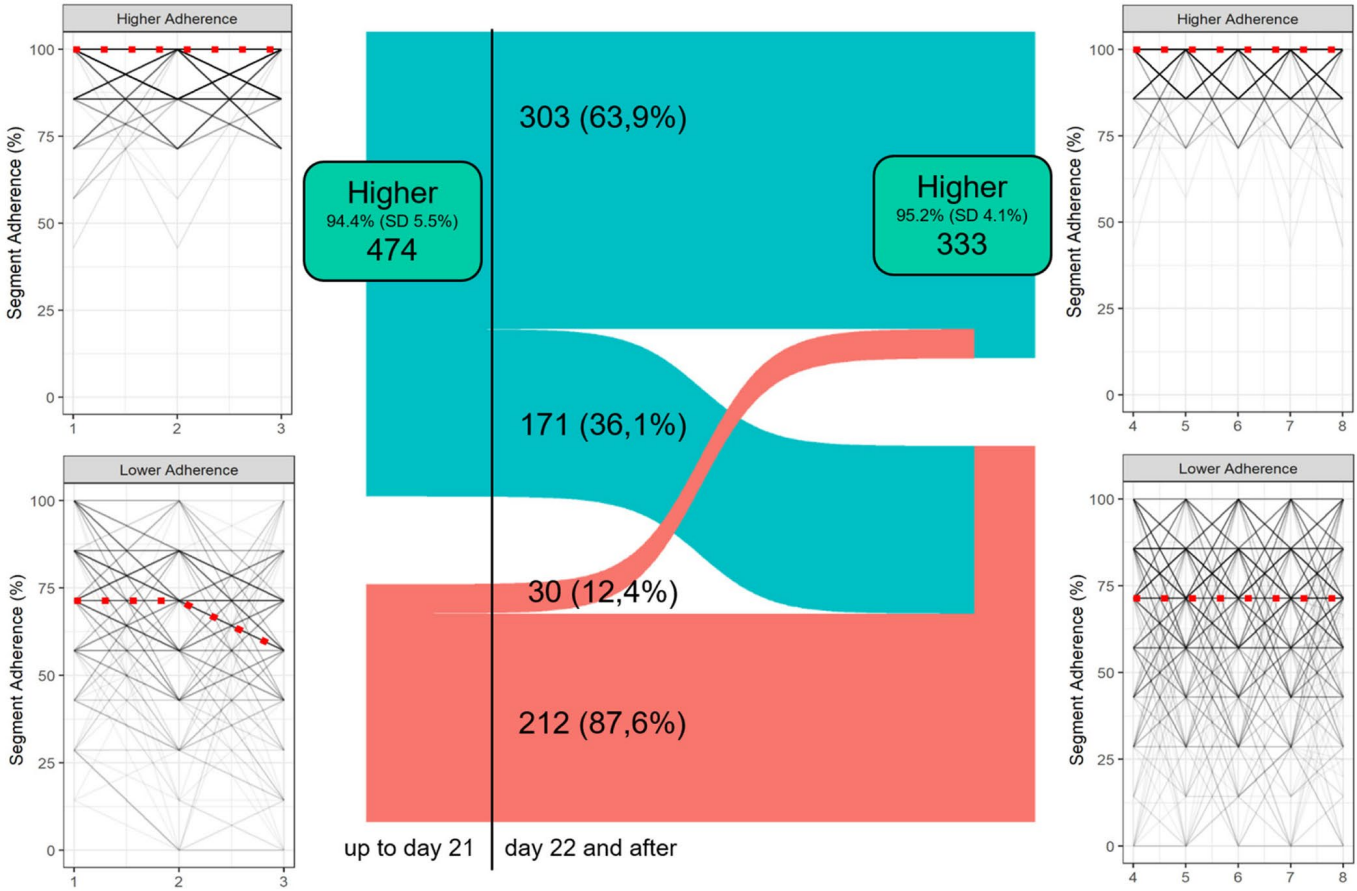
Cluster analyses with two non‐overlapping periods: Up to Day 21 (Days [1, 21]) and after Day 21 (Days [22, 56]) of the prescribed period. Side panels show the patterns of adherence over time in 7‐day intervals from the patients included in the cluster analysis grouped by cluster membership in each period. Cluster medoids are shown as red dashed lines. The ‘Higher Adherence’ cluster included 474 patients in the period before Day 21 (mean adherence 94.4%, SD 5.5%) and 333 patients in the later period (mean adherence 95.2%, SD 4.1%) and the ‘Lower Adherence’ cluster included 242 in the earlier period (mean adherence 65.1%, SD 15.8%) and 383 in the later period (mean adherence 67.1%, SD 16.3%). The alluvial plot in the middle illustrates differences in cluster membership between time periods. For differences in the proportions of patients belonging to each cluster between the study centres in both periods (Pearson's Chi‐squared test: *p* < 0.001 in both periods) see Table e3.

As adherence was not constant over time in the @IT.2020 pilot study [17], an attempt to find temporal patterns was performed by an unsupervised clustering analysis around medoids. For this analysis, each patient´s reporting behaviour was analysed in 7‐day intervals. A total of 8 consecutive 7‐day segments was considered in this analysis allowing the inclusion of 94.2% (*n* = 716) of all patients with symptom records in the study app. Two separate analyses were performed with two non‐overlapping periods: (i) up to the 21st day (early period), and (ii) between Days 22 and 56 (later period). The results revealed two distinct clusters in each period: in cluster 1 (early period *n* = 474, later period *n* = 333), patients exhibited a stable, high adherence (early period mean 94.4%, SD 5.5%; later period 95.2% SD 4.1%) (from now on ‘Higher Adherence’ cluster) while in cluster 2 (early period *n* = 242, later period *n* = 383) patients showed a lower and more variable adherence (early period mean 65.1%, SD 15.8%; later period mean 67.1% SD 16.3%) (from now on ‘Lower Adherence’ cluster) (Figure 3). Although there were differences in cluster membership for some patients between the two non‐overlapping periods, a total of 515/716 (71.9%) of patients were always assigned to the clusters with similar characteristics in both periods. Of high relevance, of the 242 patients belonging to the lower adherence cluster in the early period, only 30 (12.4%) moved to the higher adherence cluster in the later period. Therefore, a low short‐term adherence in the early period predicted a low adherence in the later period with 55.4% sensitivity and 91.0% specificity. More information on the e‐Diary reporting can be found in the online repository.

## 4 Discussion

Among pollen allergic patients from 9 study centres in 7 Southern European/Mediterranean countries, we observed that adherence to digital symptom recording via e‐Diary in a blended care setting was: (i) high (75.2%), independently from patient‐specific characteristics including age, gender, geographic location, and degree of digital literacy; (ii) stable at population level with > 70% of all patients reporting each day for over 60 days, including schoolchildren and adolescents; and (iii) predictable long‐term by assessing the reporting behaviour in the first 3 weeks.

### 4.1 Adherence to Daily e‐Diary Recording Is High in a Blended Care Setting

Our study shows that high levels of adherence to daily symptom recording can be achieved independently from patient characteristics and across different cultural backgrounds when regular personal contact with a healthcare professional (e.g., by e‐mail and/or phone) is involved in a blended care setting. In our cohort, this phenomenon is also true for patients who have not been using a health‐related app before. Similar trends have been described in studies using digital tools for non‐communicable diseases, in which personal involvement from the treating physician/researcher has been reported to be beneficial for compliance [11, 15]. On the other hand, studies with less involvement of healthcare staff have shown substantially lower adherence rates across several medical domains [12, 15]. Positive results on e‐Diary completion behaviour have also been published as bystander observations in randomised controlled trials (RCT) using patient‐reported outcomes [14, 30]. Furthermore, part of the supportive conditions from clinical trials can be found in a blended care approach combining the comfort of remote monitoring with personal contact with healthcare professionals [31].

### 4.2 High Levels of Adherence Can Be Maintained Over Several Months

Studies in other chronic diseases have shown mixed results with reliable reporting behaviour over time primarily described in well‐defined and severely affected patient groups who followed an interventional program in parallel [32, 33, 34]. Our results, by contrast, confirm the observations of the pilot study [17], that also patients whose life expectancy is usually not lowered by their disease and that do not participate in any interventional program are still able to report their symptoms for approximately 75% of the prescribed days over a substantial period of 60 days and longer. The fact that a low‐effort blended care approach led to a reliable cooperation by patients opens optimistic perspectives for remote monitoring also in other chronic diseases.

### 4.3 Schoolchildren and Adolescents Are Able to Actively Contribute to Their Health Monitoring

Paediatric e‐Diaries and scores have been proposed in several medical areas such as asthma care [35] and concussion headache [36]. Although adherence to symptom recording was shown to be high in these studies, their duration did not exceed 8 days. A recent study comparing the reporting behaviour of adolescents (13–17years), young adults (18–26years) and adults (> 26years) using the MASK‐Air e‐Diary showed no differences between the different age groups with an overall adherence of 30% [37]. To our knowledge, our dataset is the first to show that children and adolescents can successfully complete symptom e‐diaries over 60 days, independently from individual characteristics such as cultural background or education level.

### 4.4 Early Identification of Patients With Lower Adherence and Its Clinical Implications

Several attempts have been made to identify potential predictors for adherence to app use. Although characteristics like age [38], education level [39] and marital status [40] have been identified as possible markers in individual studies, several systematic reviews concluded that, due to methodological heterogeneity, it was not possible to identify universal markers of reporting behaviour [11, 12, 15]. Regarding baseline characteristics, our study confirms that of all assessed variables, only the retrospectively rated disease frequency and higher age were directly associated, although weakly, with a higher adherence to reporting.

This multicenter @IT‐2020 study, on the other hand, replicated positive results of our @IT‐2020 pilot study, which already demonstrated the association between long‐term reporting and adherence in the second and third weeks [17]. Early identification of patients with lower adherence to e‐Diary compilation may be crucial in precisely targeting educational interventions aimed at improving patient adherence, not only to e‐Diary compilation but to a broader disease management plan. Accordingly, past prescription‐refill behaviour, as recorded in e‐CRF, was a better predictor of medication adherence than health beliefs or other baseline characteristics in patients under a statin regimen [41]. Similarly, measures of previous adherence to chronic medications were relatively strong predictors of future adherence to newly initiated statins compared with other claims‐based measures [42]. Our study suggests that real‐time adherence monitoring via e‐Diary compilation may be an easy and feasible tool for early identification of patients who may adhere less in the future. Further studies are needed to test whether this also identifies patients with low adherence to regular medication intake. Such a tool may be essential to focus educational intervention on selected patients only, thus saving time and resources.

### 4.5 Limitations

Several limitations need to be considered for our study. First, data were not collected in a real‐life scenario but within an observational study. Real‐life studies are needed to confirm our findings. Second, study participants have been recruited in specialised allergy centres and may not be representative of the general patient population in the respective areas. Yet, we believe that the large number of patients and consistency of outcomes suggest that the recruitment bias is limited. Finally, the prescribed monitoring periods did not always match the relevant main pollen seasons. This challenge needs to be encountered with real‐time pollen monitoring to signal the optimal start of reporting.

## 5 Conclusions

A blended approach results in a continuously high adherence to e‐Diary compilation among allergic rhinitis patients, independently from individual patient characteristics. Early identification of lower adherence to e‐Diary compilation is possible and might be used for early intervention aimed at improving patients' performance. These outcomes should encourage further studies on e‐Diary monitoring of trigger‐related chronic diseases and open new opportunities for interventions to increase adherence to reporting, as well as potentially treatment adherence.

## Author Contributions

Contributes to (1) Conceptualization – major: P.M.M.; additional: S.T.; (2) Data curation – major: C.J.H.T.; additional: S.D., S.P., T.M.H., S.P., P.M.M.; (3) Formal analysis – major: C.J.H.T.; additional: U.G.; (4) Funding acquisition – major: P.M.M.; additional: S.D.; (5) Investigation – major: S.D.; additional: all the co‐authors; (6) Methodology – major: C.J.H.T., P.M.M., S.T.; additional: S.D., U.G.; (7) Project administration – major: S.D., T.M.H. and P.M.M.; (8a) Resources – major: P.M.M., S.D., S.T., A.T., M.A.B., S.A.; (8b) Patients – all the other co‐authors; (9a) Software for e‐Diary, e‐CRF – major: S.P., S.T.; additional: P.M.M.; (9b) analysis code: major: C.J.H.T.; additional: U.G.; (10a) Global supervision – P.M.M.; (10b) Field‐Work supervision: clinical, S.D.; aerobiological: A.T., M.A.B.; (11) Validation – not applicable; (12) Visualisation – major: C.J.H.T.; additional: P.M.M., U.G.; (13) Writing original draft – S.D., C.J.H.T., P.M.M.; (14) Writing – review and editing – major: P.M.M. and C.J.H.T.; additional, S.D., A.P., A.M., L.D., N.G.P., E.S.P., and all the co‐authors.

## Funding

S.A. was supported by the EAACI Fellowship Award of the European Academy of Allergology and Clinical Immunology. P.M.M. is funded by the Deutsche Forschungsgesellschaft (DFG) (MA47/2–1). This investigator‐initiated observational study has been supported by an unrestricted grant from Euroimmun Medizinische Labordiagnostika AG, Grant/Award Number: 118583. The Informatics Platform AllergyCARD and the app AllergyMonitor have been kindly provided by TPS Software Production.

## Conflicts of Interest

P.M.M. reports research support from Omron Healthcare Jp., Euroimmun, Hycor, MAD‐X, Thermo Fisher Scientific, TPS software production, personal fees from Anallergo, Omron Healthcare Jp., Hycor, speaker's fees from Euroimmun, Hycor, MAD‐X, Thermo Fisher Scientific, Allergopharma, ALK‐Abellò, Angelini, Bencard Allergie, HAL, Stallergenes, all outside the submitted work.

## Supporting information


**Data S1:** Supporting Information.

## Data Availability

This study has been conceived and planned in 2017. At that time, the dominant approach did not foresee data sharing or an open science policy. Therefore, considering the characteristics of the informed consent given by the patients and the many limitations imposed by the ethical committees of the 9 clinical centres participating in the study, the primary data collected for this study will not be shared with third parties unless this is explicitly authorised by all the ethical committees and anyhow not before 4 years after publication of the manuscript. Any correspondence on this data sharing should be directed to the principal investigator of the @IT‐2020 project and corresponding author (paolo.matricardi@charite.de).
