# Supplementary material for: Early Detection of Lower Adherence to Long‐Term e‐Diary Recording: A Checkpoint to Target Early Educational Intervention in Seasonal Allergic Rhinitis?
Source: Clin Exp Allergy. 2026 Jan 23;56(5):526–37. doi: 10.1111/cea.70203 (PMC13135879; doi:10.1111/cea.70203)
Supplement: Supplementary file 1 — Data S1: Supporting Information. [file CEA-56-526-s001.zip › cea70203-sup-0001-Supinfo01.docx]

**ONLINE REPOSITORY**

**Early detection of lower adherence to long-term e-Diary recording: a checkpoint to target early educational intervention in seasonal allergic rhinitis?**

Dramburg S^1^**^*^**, Hernandez Toro CJ^1,2*^Grittner U^2^, Tripodi S^5,6^, Arasi S^1,7^, Acar Şahin A^9^, Aggelidis X^10^, Barbalace A^11^, Bourgoin A^12^, Bregu B^13^, Brighetti MA^3^, Caeiro E^14,15^, Caglayan Sozmen S^16^, Caminiti L^11^, Charpin D^12^, Couto M^17^, Delgado L^18,19,20^, Di Rienzo Businco A^5^, Dimier C^12^, Dimou MV^21^, Fonseca JA^18,19^, Goksel O^22^, Hernandez D^23^, Hoffmann TM^1^, Jang DT^24^, Kalpaklioglu F^25^, Lame B^13^, Llusar R^24^, Makris M^10^, Mazon A^24^, Mesonjesi E^13^, Nieto A^24^, Öztürk A^26^, Pahus L^27^, Pajno G^11^, Panasiti I^11^, Papadopoulos NG^21,28^, Pellegrini E^29^, Pelosi S^8^, Pereira AM^18,19,20^, Pereira M^18,19^, Pinar NM^9^, Potapova E^1^, Priftanji A^13^, Psarros F^30^, Sackesen C^31^, Sfika I^5^, Suarez J^32^, Thibaudon M^33^, Travaglini A^3,4^, Uguz U^34^,Verdier V^12^, Villella V^5^, Xepapadaki P^35^, Yazici D^36,37^, and Matricardi PM^1,38,39^

^*^ both authors contributed equally to the manuscript

Author Affiliations are available in the main text.

**Supplementary Text:**

**Methods**

*Inclusion and Exclusion Criteria*

Patients had to fulfil the following inclusion criteria to participate in the study: i) diagnosis of seasonal allergic rhinitis (hay fever) ii) age 10 to 18 years for children or 19 to 60 years for adults; iii) a good understanding of the national language or one of the languages offered in the mobile study application (AllergyMonitor^TM^, TPS software production, Rome, Italy); iv) access to a smartphone; v) written informed consent. In addition, the following exclusion criteria were applied: i) prior allergen immunotherapy for pollen allergies; ii) any severe chronic disease mimicking symptoms of allergic rhinitis; iii) home address further than 30 km away from the local aerobiological centre´s pollen trap.

*Study visits*

During the recruitment visit (T0), every participant or his/her legal guardian completed a questionnaire on sociodemographic variables, clinical history of AR and asthma, family history, allergic comorbidities, and current treatment scheme. Skin prick test (SPT) and IgE tests for a panel of locally relevant allergen extracts and molecules were performed as previously described (see reference 20 of the main text). Based on the individual clinical history and SPT results, up to two periods for symptom monitoring were prescribed per participant, matching the expected pollination periods of potentially relevant allergen sources. At the follow-up visits (T1), the patient answered clinical questionnaires evaluating symptoms during the past main pollen season, usability of the e-Diary, and a validated questionnaire on digital health literacy.

*Questionnaires*

During the enrolment visit, severity and frequency of AR symptoms was assessed with the validated ARIA (Allergic Rhinitis and its Impact on Asthma) questionnaire^1^. Patients were asked to answer its questions thinking of the past pollen season. The same tool was then used during T1 to retrospectively evaluate the pollens season 2018. Severity and control of allergic asthma was measured according to the Global Initiative for Asthma (GINA) guidelines at T0 and T1^2^. Digital health literacy was assessed via the validated Digital Health Literacy Instrument (DHLI) covering (1) operational skills, to use the computer and Internet browser, (2) skills, to navigate and orientate on the Web, (3) information searching skills, (4) the ability to evaluate reliability and relevance of online information, (5) the ability to add self-generated content to Web-based apps, and (6) skills to protect and respect privacy while using the Internet. All items were evaluated on a 4-point skill with a rating of 4 indicating the highest level of literacy^3^. To evaluate the usability and user-friendliness of the AllergyMonitor^TM^ app, we used the System Usability Scale by John Brooke^4^.

*AllergyMonitor*^TM^ *e-Diary App*

The daily questionnaires in the AllergyMonitor^TM^ study app included organ-specific symptom questions based on Likert scales regarding the eyes, nose, and lungs. In addition, the medication was individually set up by the attending physician via a back-office dashboard, which allows the monitoring of the reporting behaviour and communication with the user. Patients were able to report symptoms for the current and, if not already filled, also for the previous day. The symptom and medication data collection was based on previously validated scores, such as the rhinitis total symptom score^5^ (RTSS) and the combined symptom and medication score^6^ (CSMS). In addition, the user is asked whether the allergy symptoms impacted his/her sleep, daily activity, and/or school/work productivity. Finally, the overall quality of life regarding allergy symptoms is estimated via a continuous VAS, and the medication intake is recorded.

*Definitions for adherence to symptom reporting*

To describe the reporting behaviour of our cohort, we propose a uniform terminology that has been previously published ^(reference^ ^17 of the main manuscript)^ **[Figure e1]**. While the “prescribed period” includes all days fixed by the attending physician, the “reporting period” excludes the delayed reporting start with missing days potentially due to technical problems and an anticipated reporting end potentially due to fatigue. Within the reporting period, there may be fragments with missing records of different lengths. Population level adherence is the percentage of patients reporting symptoms on a specific day out of all patients expected to be reporting their symptoms.

*Statistics*

*Estimated differences in adherence between groups of pediatric patients (who filled e-Diary)*

In the case of pediatric patients, the data input was not always performed by the patients themselves. Symptom registration may have been done together with the parent or guardian or by the parent/guardian alone. To evaluate if the person filling most of the e-Diary records was associated with the adherence in the prescribed period, a linear mixed model with center as random intercept, the adherence in prescribed period as dependent variable, and who filled the e-Diary as independent variable was used. No adjustment for other covariates was considered. Estimated marginal mean adherences in the prescribed period of the groups of pediatric patients defined by who filled the e-Diary were estimated as well as the differences between the groups with 95% confidence intervals.

*Estimated differences in adherence between ARIA classifications of AR at T0:*

Marginal Means for each level of the ARIA classification at T0 were estimated from the linear mixed model (LMM) of long-term adherence on short-term adherence, adjusted by baseline covariates (age, gender, AR ARIA classification at T0, length of prescribed period, top 4 most frequent allergic comorbidities, years of schooling, education level, DHLI, and previous use of health apps), with random intercept by study centre. Differences between these estimated means and their 95% confidence intervals were also estimated.

*Per-segment adherence clustering analysis*

To find temporal patterns in the reporting behaviour, unsupervised clustering analyses around medoids (a more robust version of k-means) were performed^7^. Each patient´s reporting behaviour was analysed in segments of 7 days (i.e., adherence between day 1 and day 7, between day 8 and day 14, etc.). A total of 8 consecutive 7-day intervals were considered allowing us to include 94% (n=716) of all patients with symptom records in the study App. The reports of excluded patients were shorter than 8x7=56 days. The optimal number of clusters was determined as the k=[2,10] that maximizes the average normalized difference between the average dissimilarity of each observation with all observations belonging to the closest cluster and the average dissimilarity of each observation with all other observations belonging to the same cluster (maximum average silhouette width)^8^.

Two separate analyses were performed with two non-overlapping subsets of 7-day intervals: i) First three intervals corresponding to days [1,21] (“early period”); ii) from the fourth to the eight intervals corresponding to days [22, 56] (“later period”). Mean and standard deviation of per-segment adherence was estimated for each cluster in each period. The frequencies of cluster membership between the two periods were described in a contingency table and an alluvial plot.

To identify potential risk factors associated with lower adherence in the later period within the subpopulation of patients belonging to the “Higher Adherence” cluster in the early period (n=383), we performed a logistic regression analysis (optimal number of clusters was determined as 2) with study centre as random intercept and baseline characteristics (age, gender, AR ARIA classification at T0, length of prescribed period, top 4 most frequent allergic comorbidities, education level, years of schooling, DHLI, previous use of health apps) and who filled the e-Diary as independent variables. Odds ratios and partial R^2^ were estimated for all covariates included in the model. The association of study centre with cluster membership was evaluated with a Chi-Square test in each of the non-overlapping periods considered (“early” and “later” periods).

*Statistical software*

All statistical analyses were conducted using R (version 4.2.2) (<http://www.r-project.org/>). The 'tableone' (version 0.13.2)^9^ library was used to generate summary statistics and estimate mean SMD, 'lme4' (version 1.1.34 ^10^ to fit linear and generalized linear mixed-effects models, ‘sjPlot’ (version 2.8.15)^11^ to create mixed-effects models summary tables, ‘emmeans’ (1.8.5)^12^ to estimate marginal means of covariates in linear models, ‘r2glmm’ (version 0.1.2) ^13^ to estimate partial R squared, and 'cluster' (version 2.1.4)^14^ to perform clustering analyses, ‘ROCR’ (version 1.0.11)^15^ to estimate Area Under ROC Curve, ‘caret’ (version 6.0.94)^16^ to estimate metrics from confusion matrices, ‘ggpmisc’ (version 0.5.6)^17^ to estimate Pearson and Spearman correlation in bivariate plots, and ‘ggsankey’ (version 0.0.99999)^18^ to generate alluvial plots.

*Role of the funding source*

The field work of the @IT-2020 pilot and multicenter projects have been supported by an unrestricted grant from Euroimmun Medizinische Labordiagnostika AG (Lübeck, Germany) which has played no additional role in the conduct of the project and in the preparation of this manuscript.

**Results**

*Baseline characteristics*

Of the entire study population, 54.1% (441/815) were male with no major differences between study centers (SMD: 0.12). As not all study centers recruited the same number of patients for each age group (e.g. Marseille only included adults), differences regarding the participants’ age, disease duration, and level of education were observed between study centers. While most centers had a high proportion of patients filling the e-Diary (92.8-100.0%), Valencia is the exception with only 33/71 (46.5%) of reporting participants **[Table e1].**

The number of serologically and clinically relevant seasonal airborne allergens ranged from a predominance of a single allergen (e.g., Parietaria pollen in Messina, Italy) to high rates of poly-sensitized patients (e.g., in Rome, Italy or Tirana, Albania) **[Table e1]**. Despite this diversity, grass pollen allergens were among the most frequent triggers of hay fever symptoms in all study centers except in Messina, Italy.

Adherence to e-Diary symptom reporting

The average length of monitoring periods prescribed by the treating doctors differed by center, keeping in line with the variable duration and multiplicity of the local airborne pollen seasons^19^ **[Table e4]**. Longer monitoring periods reflect a higher number of clinically relevant pollen allergens which makes an extended observation necessary to cover the pollination seasons of all potentially relevant sources of airborne allergen triggers. On average, 1.1% (SD: 3.8%) of the records were missed due to a delayed reporting start and 4.9% (SD: 12.8%) due to an anticipated reporting end **[Table 1]**.

Larger variations in adherence after 60 days of reporting may be explained by the decreasing number of subjects with increasing length of the prescribed monitoring period, **[Figure e2].** Adherence to e-Diary compilation was not significantly influenced by the level of exposure to airborne pollen (data not shown), but to symptom severity (RTSS) during the previous 5 days **[Figure e3, Table e5]**. However, the effect size of this association is very small, as reflected in the model slope (0.29) and small Marginal R^2^ (0.001).

*Association between short-term reporting behavior and long-term adherence*

Despite the observed positive correlation between short- and long-term adherence in all centres, some differences occurred between centres in terms of the strength of association. While the short-term behaviour correlated more strongly with long-term recording adherence in Istanbul, Izmir, Marseille, Messina, and Porto (R and/or ρ > 0.65), this association was weaker in Athens (R=0.59, ρ=0.57), and weakest in Rome, Tirana, and Valencia (R or ρ ≤ 0.50) **[Figure 2]**.

Despite no substantial associations between baseline characteristics and long-term adherence, we observed a small effect of disease severity. Patients who reported persistent symptoms in the ARIA questionnaire at T0 had on average higher long-term adherence than patients reporting mild intermittent symptoms. However, no differences between patients reporting moderate to severe intermittent symptoms at T0 compared to all others ARIA classes were found **[Table e6].**

From the potential risk factors associated with a lower adherence in the later period (membership to “Lower Adherence”) in the subpopulation of patients belonging to the “Higher Adherence” cluster in the early period, only the retrospectively estimated symptom severity and frequency (ARIA classification) at T0 was identified as significant, indicating that patients who reported being more severely and/or more frequently affected by symptoms of the disease at T0 tended to exhibit higher adherence. The logistic regression used in this analysis had an Area under ROC Curve of 0.63, and a Brier Score of 0.21. **[Table e7]**.

**References:**

1. Brozek JL, Bousquet J, Baena-Cagnani CE, Bonini S, Canonica GW, Casale TB, Global Allergy and Asthma European Network, Grading of Recommendations Assessment‚ Development and Evaluation Working Group. Allergic Rhinitis and its Impact on Asthma (ARIA) guidelines: 2010 revision. J Allergy Clin Immunol 2010;126:466-476. doi: 10.1016/j.jaci.2010.06.047
2. <https://ginasthma.org/2023-gina-main-report/> (last accessed on 24 January 2024)
3. van der Vaart R, Drossaert C. Development of the Digital Health Literacy Instrument: Measuring a Broad Spectrum of Health 1.0 and Health 2.0 Skills. J Med Internet Res. 2017 Jan 24;19(1):e27. doi: 10.2196/jmir.6709.
4. Brooke J. SUS: A quick and dirty usability scale. Usability Eval Ind. 1995;189.
5. Devillier P, Chassany O, Vicaut E, de Beaumont O, Robin B, Dreyfus JF, Bousquet PJ. The minimally important difference in the Rhinoconjunctivitis Total Symptom Score in grass-pollen-induced allergic rhinoconjunctivitis. Allergy. 2014 Dec;69(12):1689-95. doi: 10.1111/all.12518. Epub 2014 Oct 6.
6. Pfaar O, Demoly P, Gerth van Wijk R, Bonini S, Bousquet J, Canonica GW, Durham SR, Jacobsen L, Malling HJ, Mösges R, Papadopoulos NG, Rak S, Rodriguez del Rio P, Valovirta E, Wahn U, Calderon MA; European Academy of Allergy and Clinical Immunology. Recommendations for the standardization of clinical outcomes used in allergen immunotherapy trials for allergic rhinoconjunctivitis: an EAACI Position Paper. Allergy. 2014 Jul;69(7):854-67
7. Kaufman, L. and Rousseeuw, P.J. (1990). Partitioning Around Medoids (Program PAM). In Finding Groups in Data (eds L. Kaufman and P.J. Rousseeuw). <https://doi.org/10.1002/9780470316801.ch2>
8. Rousseeuw PJ. Silhouettes: A graphical aid to the interpretation and validation of cluster analysis. Journal of Computational and Applied Mathematics. 1987;20:53-65.
9. Yoshida K, Bartel A (2022). _tableone: Create 'Table 1' to Describe Baseline Characteristics with or without Propensity Score Weights_. R package version 0.13.2, <https://CRAN.R-project.org/package=tableone>
10. Douglas Bates, Martin Maechler, Ben Bolker, Steve Walker (2015). Fitting Linear Mixed-Effects Models Using lme4. Journal of Statistical Software, 67(1), 1-48. doi:10.18637/jss.v067.i01.
11. Lüdecke D (2023). _sjPlot: Data Visualization for Statistics in Social Science_. R package version 2.8.15, <https://CRAN.R-project.org/package=sjPlot>.
12. Lenth R (2023). _emmeans: Estimated Marginal Means, aka Least-Squares Means_. R package version 1.8.5, <https://CRAN.R-project.org/package=emmeans>.
13. Jaeger B (2017). _r2glmm: Computes R Squared for Mixed (Multilevel) Models_. R package version 0.1.2, <https://CRAN.R-project.org/package=r2glmm>.
14. Maechler, M., Rousseeuw, P., Struyf, A., Hubert, M., Hornik, K.(2022). cluster: Cluster Analysis Basics and Extensions. R package version 2.1.4.
15. Sing T, Sander O, Beerenwinkel N, Lengauer T (2005). “ROCR: visualizing classifier performance in R.” Bioinformatics, 21(20), 7881. <http://rocr.bioinf.mpi-sb.mpg.de>.
16. Kuhn, M. (2008). Building Predictive Models in R Using the caret Package. Journal of Statistical Software, 28(5), 1–26. <https://doi.org/10.18637/jss.v028.i05>
17. Aphalo P (2024). ggpmisc: Miscellaneous Extensions to 'ggplot2'. R package version 0.5.6, <https://CRAN.R-project.org/package=ggpmisc>.
18. Sjoberg D (2023). ggsankey: Sankey, Alluvial and Sankey Bump Plots. R package version 0.0.99999
19. Hoffmann TM, Acar Şahin A, Aggelidis X, Arasi S, Barbalace A, Bourgoin A et al. "Whole" vs. "fragmented" approach to EAACI pollen season definitions: A multicenter study in six Southern European cities. Allergy. 2020 Jul;75(7):1659-1671. doi: 10.1111/all.14153. Epub 2020 May 11. PMID: 31833579.

**Legends to the Supplementary Figures**

**Figure e1 –** Graphical representation of definitions used in this study. The box line represents the monitored period (each box is a specific day) of a hypothetical participant. In this example the medical doctor, according to the individual participant’s clinical history, invited the patient to fill in the e-diary questionnaire for 54 days (prescription period). The patient started to record symptoms 6 days after the prescribed beginning day (delayed reporting start) and finished recording symptoms 5 days before the prescribed ending day (advanced reporting end). Thus, the reporting period lasted 43 days, during which the participant did not fill in the e-diary questionnaire for 7 days (missing reporting days). Overall, the participant filled in the e-diary questionnaire for 36 days (reporting days). (From Di Fraia M, et al. JMIR 2020, reproduced with permission)**.**

**Figure e2 -** Population level adherence to symptom recording in study app (N=760) per center within A) reporting periods and B) prescribed periods. Curves were smoothed with spline models with 25 degrees of freedom. Percentage of patients expected to report (i.e., day within prescribed period) and of active patients (i.e., day within reporting period) at each time point per center are shown as dashed lines.

**Figure e3 –** Association of RTSS of the previous 5 days (including reference day) with adherence in the following 5 days (non-overlapping periods).


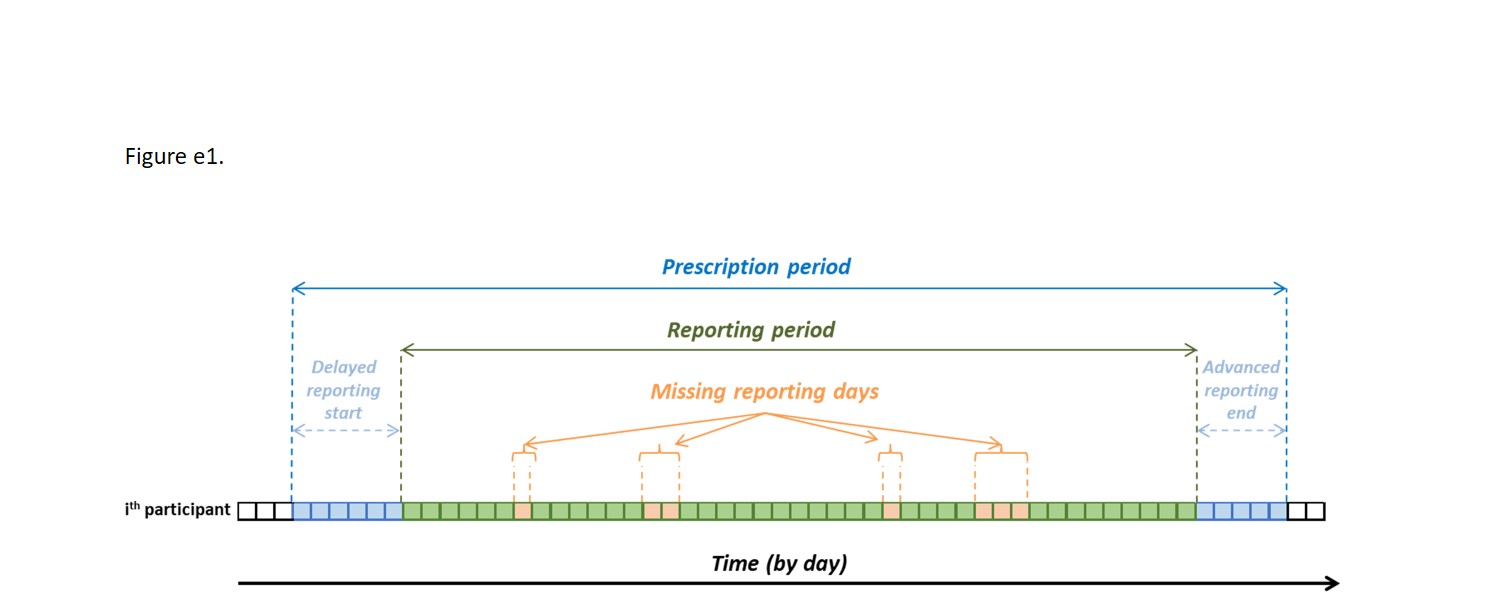


**Figure e1**


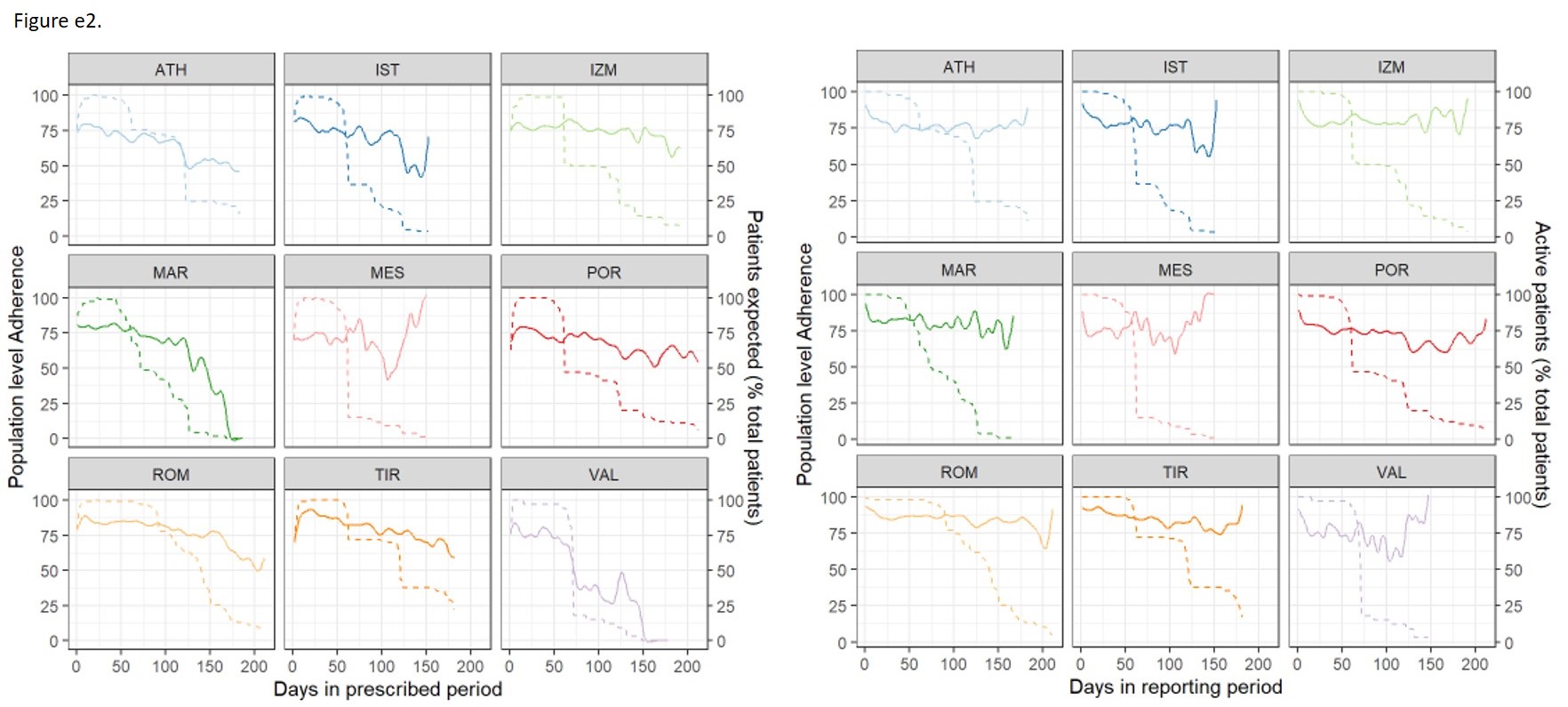


**Figure e2**


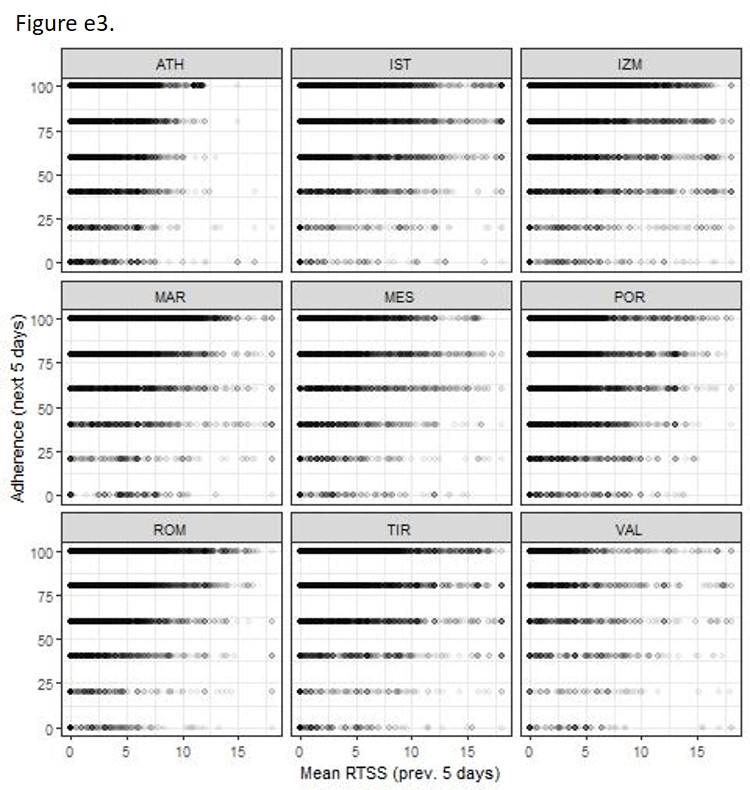


**Figure e3**

**Legends to the Supplementary Tables**

**Table e1 -** Baseline and T1 characteristics of the study population by study centre.

**Table e2 -** Estimated marginal mean adherence in the prescribed period of pediatric patients according to who filled the e-Diary and the differences between these. Estimates from LMM (random intercept by centre) with "who filled e-Diary" as independent and adherence in prescribed period as dependent variables.

**Table e3 –** Absolute frequency per center of "per segment adherence" cluster membership in each time period. Statistical significance of this association within each period was tested with Pearson's Chi-squared test.

**Table e4** - Overview of the prescribed reporting periods per allergen source and center

**Table e5** - Linear mixed model on the influence of symptom severity (mean RTSS) in the previous 5 days on mean adherence in the future 5 days, with a random intercept at the patient level and without adjusting for other confounders.

**Table e6** - Estimated marginal mean long-term adherence in the prescribed period of patients per ARIA classification of AR at T0 and differences between these. Estimates from LMM adjusting for age, short-term adherence, education level, years of schooling, DHLI, and previous use of health mobile apps (n=568)

**Table e7** - Associations of baseline characteristics with cluster membership in the later period of the patients assigned to "High Adherence" cluster in the earlier period estimated from logistic mixed mod
